# Effect of cross-platform variations on transthoracic echocardiography measurements and clinical diagnosis

**DOI:** 10.1093/ehjimp/qyae097

**Published:** 2024-09-23

**Authors:** Mohammad Saber Hashemi, Yasaman Farsiani, Gregg S Pressman, M Reza Amini, Arash Kheradvar

**Affiliations:** Department of Biomedical Engineering, University of California Irvine, Irvine, CA, USA; Department of Biomedical Engineering, University of California Irvine, Irvine, CA, USA; Division of Cardiology, Thomas Jefferson University, Thomas Jefferson Einstein Hospital, Philadelphia, PA, USA; Section of Cardiology, Department of Medicine, Loma Linda University, Loma Linda, CA, USA; Department of Biomedical Engineering, University of California Irvine, Irvine, CA, USA; Mary & Steve Wen Cardiovascular Division, Department of Medicine, University of California Irvine, Orange, CA, USA

**Keywords:** transthoracic echocardiography, three-dimensional echocardiography, reproducibility, left ventricle, right ventricle, ejection fraction, volumes, cross-platform variation

## Abstract

**Aims:**

Accurate cardiac chamber quantification is essential for clinical decisions and ideally should be consistent across different echocardiography systems. This study evaluates variations between the Philips EPIQ CVx (version 9.0.3) and Canon Aplio i900 (version 7.0) in measuring cardiac volumes, ventricular function, and valve structures.

**Methods and results:**

In this gender-balanced, single-centre study, 40 healthy volunteers (20 females and 20 males) aged 40 years and older (mean age 56.75 ± 11.57 years) were scanned alternately with both systems by the same sonographer using identical settings for both 2D and 4D acquisitions. We compared left ventricular (LV) and right ventricular (RV) volumes using paired *t*-tests, with significance set at *P* < 0.05. Correlation and Bland–Altman plots were used for quantities showing significant differences. Two board-certified cardiologists evaluated valve anatomy for each platform. The results showed no significant differences in LV end-systolic volume and LV ejection fraction between platforms. However, LV end-diastolic volume (LVEDV) differed significantly (biplane: *P* = 0.018; 4D: *P* = 0.028). Right ventricular (RV) measurements in 4D showed no significant differences, but there were notable disparities in 2D and 4D volumes within each platform (*P* < 0.01). Significant differences were also found in the LV systolic dyssynchrony index (*P* = 0.03), LV longitudinal strain (*P* = 0.04), LV twist (*P* = 0.004), and LV torsion (*P* = 0.005). Valve structure assessments varied, with more abnormalities noted on the Philips platform.

**Conclusion:**

Although LV and RV volumetric measurements are generally comparable, significant differences in LVEDV, LV strain metrics, and 2D vs. 4D measurements exist. These variations should be considered when using different platforms for patient follow-ups.

## Graphical Abstract

**Graphical Abstract:**
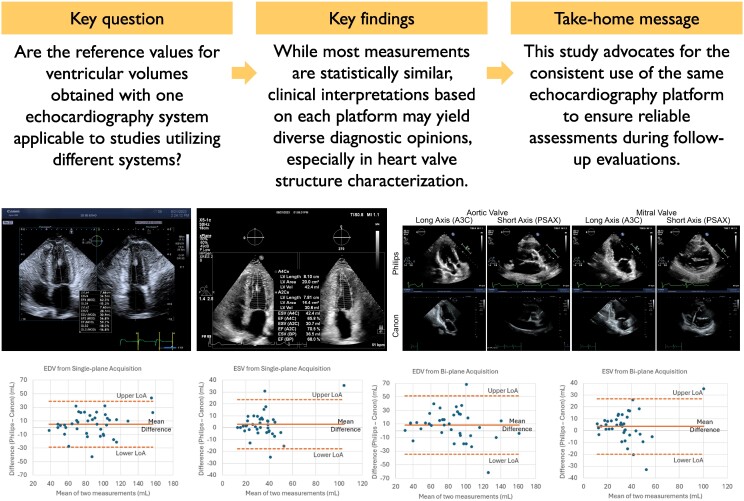

## Introduction

Transthoracic echocardiography (TTE) is the most frequently employed non-invasive cardiac procedure for routine screening purposes and is a first-line tool for clinical decision-making.^1^ It provides information on cardiac chamber size and function as well as accurate assessment of valvular function. In the USA, four primary echocardiography platforms are currently available in clinical settings: Siemens, GE, Philips, and Canon.

Numerous efforts have been undertaken to standardize the format of echocardiographic reports,^2–4^ reflecting the prevailing belief that scans conducted on any of these four platforms yield comparable imaging results.^1,3^ However, very few studies have rigorously compared these systems in a controlled setting to test this hypothesis.^5^ More importantly, several studies have highlighted inter-vendor variability in ventricular volumes and strain, raising questions about the consistency of measurements across the various platforms.^5–10^

Assessment of cross-platform variation holds significant scientific and clinical implications. Scientifically, it is crucial to determine whether reference values for ventricular volumes and ejection fraction (EF) obtained with one vendor’s system are applicable to studies utilizing different systems.^5^ Experts concur that time-dependent variations in echocardiographic findings during patient’s follow-up should be carefully considered, always prioritizing the consistency of parameters used for comparison.^2^ The question that still needs to be addressed is whether patients can be scanned for follow-up using any platform or whether it is essential to consistently use the same platform for reliable assessment for follow-up purposes, as recommended by some guidelines.^11^ Acknowledging the potential for cross-platform variation in echocardiography results is crucial for accurate diagnosis and follow-up, particularly when patients undergo scans using different platforms during their diagnosis and treatment journey.

In this prospective observational study, we examine the impact of cross-platform variations between a Philips EPIQ CVx system (version 9.0.3) and a Canon Aplio i900 system (version 7.0) on various parameters of interest within a controlled study design environment. Specifically, our objectives are to: (i) compare the cross-platform variations in 4D ventricular volumes and strain metrics of left and right ventricles measured by the same sonographer using the Philips EPIQ CVx and Canon Aplio i900 systems in the same study subjects, (ii) analyse and compare the cross-platform variability of 2D left ventricle (LV) volume and EF measurements from semi-automated methods (i.e. with manual editing by the sonographers), and finally (iii) examine whether expert assessment of valve structure varies by platform upon analysis of the data collected by the same sonographer from each study subject.

## Methods

### Study design and participants

In August 2023, we prospectively recruited 40 volunteer subjects (20 males and 20 females) aged 40 years and above for scanning at the Kheradvar Laboratory at UC Irvine. We chose a minimum age of 40 years to increase the likelihood of detecting abnormalities within the normal population. The sole criterion for recruitment, in addition to age > 40 years, was the ability to provide consent in English. An email inviting participation was dispatched to all UCI employees and students, outlining the study’s objectives and eligibility criteria. The initial cohort of 40 subjects (20 males and 20 females) was then recruited for the study. To avoid any bias, we did not inquire about the subjects’ ethnicity, race, known cardiovascular conditions, or medication. Physicians’ assessments of each subject were based solely on the echocardiography data and subject’s age available on the echocardiography images. Study participants were scheduled for echocardiography scans at Kheradvar Laboratory over an 8-day period based on their preferred schedules, with only five subjects scanned each day. Height and weight were recorded according to the subjects’ self-report, and body surface area was automatically calculated by each system. Four expert sonographers (with a minimum of 2 years of experience in routine echocardiography) conducted all scans; each was assigned 10 subjects to scan over two consecutive days. All subjects underwent sequential scanning by a Canon i-series i900 with a PSI-28VX transducer (Canon Medical Systems, Tustin, CA, USA) and Philips EPIQ CVx with an X5-1c transducer (Philips Medical Systems, Andover, MA, USA) by the same sonographer. Each subject was allocated 90 min for scanning using the two systems.

To mitigate potential bias in image acquisition across two ultrasound systems, participants underwent scans in a sequence that alternated between different systems. Specifically, on Day 1, sonographers initially used the Philips system followed by the Canon system, and on Day 2, they reversed this sequence. This alternating approach was consistently applied to all study participants throughout the 8-day imaging period.

Each study subject was assigned a unique numeric code, and batches of data were submitted to two board-certified cardiologists (M.R.A. and G.S.P.) for analysis and reporting. We first submitted the batch of data from one vendor for analysis, and after completion of analysis and reporting for all 40 datasets, the data batch from other vendor was provided to both MRA and GSP for analysis and reporting a week later. When working on each data set, MRA and GSP did not go back to the other vendor’s batch. Therefore, every echocardiographic study was analysed uniquely and independently.

### Image acquisition and analysis

All subjects were examined on a standard medical examination bed, positioned in an appropriate orientation during the left and right ventricle scanning. Sonographers were tasked with acquiring high-quality standard 2D and 3D echocardiographic data. Optimal machine settings and image acquisition in accordance with the respective manufacturers’ recommendations were applied. All data were stored in the standard Digital Imaging and Communications in Medicine (DICOM) format to enable post-processing with independent software packages.

#### 2D Image acquisitions

**Figure 1:**
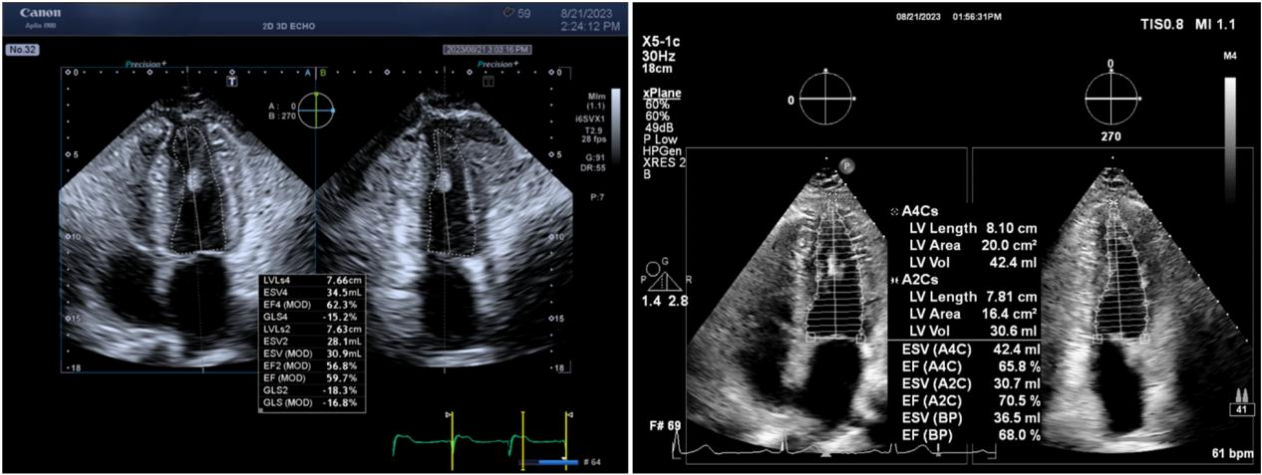
A visual comparison between the Canon and Philips platforms. The image presents an example of 2D biplane analysis results by Canon (left image) alongside results from the same subject obtained using the Philips platform (right image).

The acquisition protocol aimed to ensure consistent and reproducible views of the apical 4-chamber (A4C) and apical 2-chamber (A2C) views for analysing and comparing results of echocardiographic method of disk (MOD) Simpson’s EF, and MOD end-diastolic volumes (EDV), MOD end-systolic volumes (ESV). At least three consecutive cycles were recorded for each view. The digital acquisition of images was conducted in 2D greyscale imaging. System settings were adjusted to enhance the clarity of the ventricular myocardium, facilitating comprehensive analysis during both diastole and systole for the left ventricle (LV). ECG optimization aimed to minimize artefacts, ensuring consistent timing for analysis across two distinct ultrasound systems. The width and depth of the ultrasound 2D sectors were individually optimized and matched for A4C and A2C views in each scan. Notably, during acquisition, the apical views extended 1 cm beyond the left atrial wall (*Figure 1*).

Ensuring uniformity in acquired images and views across two distinct ultrasound systems involved a careful review of previously captured images. Each sonographer thoroughly compared and aligned clinical markers in each image, focusing on critical elements such as the septal wall and lateral wall. This included the position, size, and location of papillary muscles, LV apex, and ensuring accurate A4C and A2C views without aortic valve presence. Optimization measures were implemented to avoid any foreshortening or extension of the LV image (*Figure 1*).

In our study, the term ‘single-plane assessment’ refers to the acquisition method where the volume is calculated using separate 2-chamber (2ch) and 4-chamber (4ch) image sets. These images are acquired independently, and the volume is determined by combining them using the summation of elliptical disks (Simpson’s method). In contrast, the ‘biplane assessment’ involves acquiring both 2ch and 4ch images simultaneously using a biplane acquisition technique. Although the volume calculation method remains the same (stacked elliptical disks), the 2ch and 4ch images are obtained concurrently rather than separately.

##### 2D Image processing

To compute volumes on each system, A2C and A4C views of the heart were obtained through single-plane acquisitions and biplane acquisitions where A2C and A4C views were simultaneously collected. The Simpsons’ biplane method using elliptical disks was utilized for volume calculation. Crucial information required for determining volumes and EF includes the endocardial border contour at end-systole (ES) and end-diastole (ED), along with the corresponding frames of those timepoints.

In single-plane acquisitions of the A2C and A4C images on the Canon system, the Auto EF feature was used for volume calculations. When opening the image clip of the A2C or A4C view, the platform’s algorithm automatically estimates the endocardial border using advanced segmentation and active contouring methods, providing a contour line that can be adjusted at the estimated ES and ED. The operators have the flexibility to manually adjust this contour line outward or inward from its starting position to align with their perception of the endocardial border. The closer the operator places the curser on the editable contour line, the smaller the region of adjustment, i.e. fewer control points on the contour line will move. Additionally, operators could edit the ED and ES timepoints. After analysing both A2C and A4C clips (single-plane acquisition), the EDV and ESV are calculated using the Simpson’s biplane method (elliptical disks).^12^

In the biplane acquisition clip within the Canon system, upon opening the clip, both A2C and A4C views are displayed. The user proceeds to select the frame they identify as ES and manually traces the endocardium on both A2C and A4C view images. Subsequently, the operator scrolls to ED and repeats the tracing process. Once the endocardial borders are traced and the contour lines accepted, the ESV and EDV are calculated according to the Simpsons’ biplane method.

For both single-plane and biplane acquisitions on the Philips platform, the operator manually determines the ES and ED to establish the endocardial border contour. The technique to generate the contour for either data set is consistent such that the user initially selects the frame representing ED and marks the two insertion points of the mitral valve, resulting in the creation of a third point for a contour model. This third point is then moved by the operator to the LV apex forming the contour line. The user can adjust the contour line inward or outward using a set of control points along its length. Once the operator accepts the contour placement at the ES and ED, the EDV and ESV are calculated using the Simpsons’ biplane method, which is identical to the method used on the Canon platform.

#### 4D Image acquisitions

The protocol was aimed to ensure consistent and reproducible 4D volumetric data with the goal of analysing 4D volumes and 4D strain. There were several key steps on the initial digital image preparation that were conducted in 2D greyscale imaging prior to acquisition of 4D imaging. The first step was to visualize the A4C using 2D imaging aiming to align the probe to get the same A4C view plane as the 2D acquisition. This is to enhance the clarity of the ventricular myocardium throughout the cardiac cycle. The ECG was optimized to minimize artefacts, ensuring consistent timing for analysis across two distinct ultrasound systems. The width and depth of the ultrasound 2D image were individually optimized and matched for the A4C view. Notably, during acquisition, the apical views extended 1 cm beyond the left atrial wall.

##### 4D LV image acquisition protocol

With the imaging of the A4C view optimized as discussed earlier, the 4D image rendering was triggered by creating a 4D ‘full volume’ 70^○^×70^○^ data set with a matching depth for each comparable study. Image visualization and acquisition of the live 4D image include the whole LV and left atrial myocardium.

To ensure uniformity of the acquired images and views for each study participant across the two distinct ultrasound systems, each sonographer performed a careful review of the previously acquired images to help setup the proper probe orientation for the 4D acquisition. The sonographer thoroughly compared and aligned clinical markers in the A4C image, focusing on critical elements such as the septal and lateral walls. This involved documenting the position, size, and location of papillary muscles and the LV apex, while also ensuring consistency and accuracy across the 4D data sets for each platform’s images. Additionally, optimization measures were implemented to avoid any foreshortening or extension of the LV images during the 4D imaging.

##### 4D RV image acquisition protocol

The probe orientation and alignment of the RV chamber within the A4C view plane was matched for both systems to ensure acquisition of similar width and depth of the ultrasound 2D images. Once the A4C view was optimized, the 4D imaging was initiated, generating a 4D ‘full volume’ data set with consistent depth and width for each comparable study, with a specific focus on RV and right atrium (RA).

To achieve consistency in the acquired images for each study participant using two separate ultrasound systems, a thorough examination of previously captured images was conducted. Each sonographer carefully reviewed and aligned clinical markers in the A4C RV and RA images, with a specific focus on crucial elements, e.g. septal wall, RV free wall, and RA. This process guaranteed accurate and uniform views across the 4D data sets for the images captured by each platform. Additionally, optimization measures were implemented to prevent any foreshortening or extension of the RV image during the 4D image acquisition.

##### 4D Image processing

TomTec-Arena (TTA) Ultrasound Workspace (Philips Medical Systems) was used for post-processing of 3D measurements. All measurements were taken by a single observer (Y.F.). For each DICOM file, a trained operator (Y.F.) adjusted the reference points for 4D tracking of the ventricles’ borders ensuring volumetric segmentation. TTA Ultrasound Workspace is widely used clinically for post-processing of 4D echocardiography, and it is presumed to work cross-platform. The TTA’s 4D LV-ANALYSIS tool generates a 4D model (Beutel) of the LV cavity, which is used to measure the global and segmental strain and displacement. The application automatically conducts LV view alignment, ES, and ED timepoints estimation, LV tracking revision, and analysis, respectively. Nevertheless, users retain the flexibility to manually adjust the software’s calculations within the graphical user interface as needed. For instance, the apex axis endpoints, as well as the mitral valve and other landmarks, can be repositioned via drag-and-drop functionality to align with the reference diagram. Additionally, the dynamic 3D surface model of the ventricle, Beutel, can be adjusted through contour editing at the ED and ES. Upon conducting analysis, various parameters along with the volume curve are calculated and extracted. The process for 4D RV-Function differs slightly, as it involves a semi-automatic generation of the 3D surface model of RV, i.e. the user is required to manually adjust landmarks like the RV apex and tricuspid valve locations during view adjustment, and to refine the application’s tracing of the RV borders. Following these adjustments within the software would yield the results.

### Assessment of the heart valves

Valve morphology was assessed by examining each valve from multiple imaging views: PLAX, PSAX, and APLAX for the aortic valve; PLAX, PSAX, A4C, A2C, APLAX, and subcostal views for the mitral valve; and RV inflow, A4C, RV focused, and subcostal views for the tricuspid valve. Valve leaflets were deemed normal if they appeared thin and were freely mobile. Abnormalities were identified if there was focal or diffuse thickening, a heterogeneous appearance with areas of increased echogenicity suggesting sclerotic changes, or reduced leaflet mobility. Assessments were performed by two independent board-certified cardiologists (M.R.A. and G.S.P.). The grading of the valves was based solely on echocardiographic images. To avoid bias, we did not inquire about the subjects’ known cardiovascular conditions or medications. We applied consistent standards for evaluating the heart valves across both systems.

### Statistical analysis

Continuous variables were summarized as mean ± standard deviation ($\mu_{0}\pm \sigma$) or as median (95% confidence interval), whereas scalar variables were reported as percentages. All analyses were performed using Excel (Microsoft Corporation, Redmond, WA, USA) and MATLAB R2023b (MathWorks, Natick, MA, USA). To confirm the assumption of data normality, the one-sample Kolmogrov–Smirnov test (with a significance level of 5%) was performed on the 4D LVEF data. The test failed to reject the null hypotheses, i.e. the samples are presumably coming from normal distributions, with the *P*-values of 0.83 and 0.74 for the Philips and Canon acquisitions, respectively. Upon confirmation of normality, paired sample *t*-tests were performed on measurements from both Canon and Philips systems. A two-tailed *P*-value <0.05 indicates statistical significance, suggesting that the measurements from both systems differ significantly and are unlikely to be attributed to chance or simple randomness. For variables showing significant differences, we included the correlation and Bland–Altman plots. The correlation coefficient $R^{2}$, which is the squared Pearson correlation coefficient, measures the direction and strength of a linear relationship between two variables. This unitless measure ranges from −1 to 1, where −1 indicates a perfect inverse correlation, 0 indicates no linear correlation, and 1 indicates a perfect direct correlation. Considering *x* and *y* as paired random variables sampled from a population, with *n* data pairs and $\bar{x}$ representing the mean of the sampled *x* values, the coefficient is defined as:


$$R^{2}=\frac{\sum_{i=1}^{n}\;(x_{i}-\bar{x})(y_{i}-\bar{y})}{\sqrt{\sum_{i=1}^{n}\;{\left(x_{i}-\bar{x}\right)}^{2}}\sqrt{\sum_{i=1}^{n}\;{\left(y_{i}-\bar{y}\right)}^{2}}}$$

A Bland–Altman plot illustrates the level of agreement between two measurement methods by plotting each sampled pair of measurements with the *x*-value representing their mean and the *y*-value representing their difference. To detect any systematic differences (i.e. fixed bias), the mean difference (estimated bias) is plotted as $y=\bar{\Delta }$. To identify possible outliers or proportional biases, the 95% limits of agreement (LOAs) are plotted as $y=\bar{\Delta }\pm 1.96\;SD(\Delta_{i})$, where SD denotes the standard deviation.

### Power analysis

To determine an appropriate sample size for our study, which aims to identify potential differences between the two platforms, we focused on the key quantities of interest (QoIs): LVEF, RVEF, GLS (for LV), and GCS (for LV). These measures are crucial in clinical diagnosis and directly measured in our study. We conducted a power analysis to ensure that our sample size is sufficient to detect a meaningful difference of at least 10% in the mean values of all QoIs. According to global statistics,^13,14^ the mean ± SD for these measures are as follows: LVEF: 61 ± 5 (%); RVEF: 56 ± 5 (%); GLS: −21 ± 3 (%); and GCS: −32 ± 4 (%). Assuming a normal distribution for the variables in our experiment and using α = 0.05 and β = 0.10 (equivalent to 90% power) for hypothesis testing, with effect sizes of 1.22, 1.12, 0.70, and 0.80, the minimum sample size that satisfies all four measures was calculated to be 22. To ensure sufficient power for comparing the two platforms across all four measures, we selected a sample size of 40.’

## Results

### Study population

A total of 40 subjects were consecutively screened for inclusion in the study, and there were no dropouts. 2D and 3D LV and RV quantification using the two echocardiography systems were successfully performed in all 40 subjects. Patients’ mean age and body mass index were 56.75 ± 11.57 and 26.08 ± 5.48, respectively. The total time allocated for each subject to be scanned by both systems was 90 min, and all scans were completed within 90 min or less. Heart rate was found comparable between the two paired studies (biplane: *P* = 0.19; single-plane: *P* = 0.13).

### Conventional 2D analysis of LV

**Figure 2:**
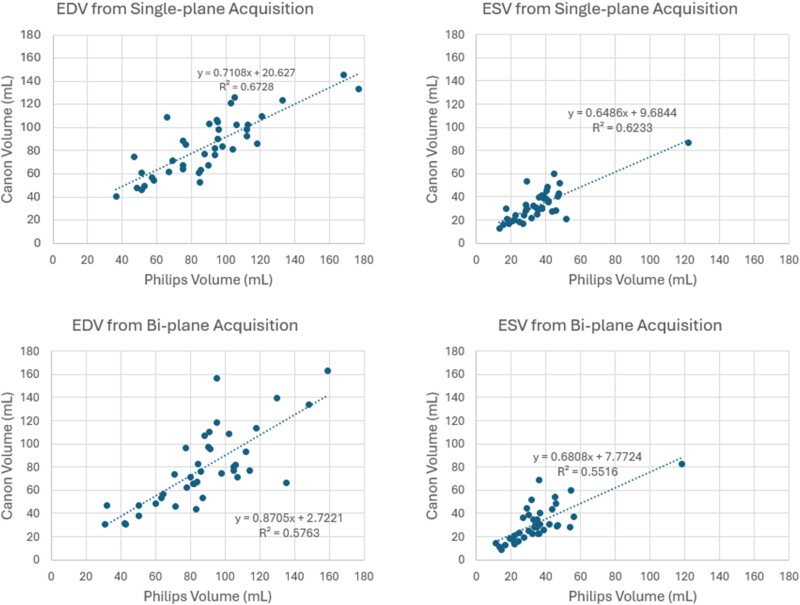
Cross-platform correlation plots compare the 2D-based calculation of ventricular volumes at ED and ES. A comparison between biplane and single-plane is provided. EDV, end-diastolic volume; ESV, end-systolic volume.

**Figure 3:**
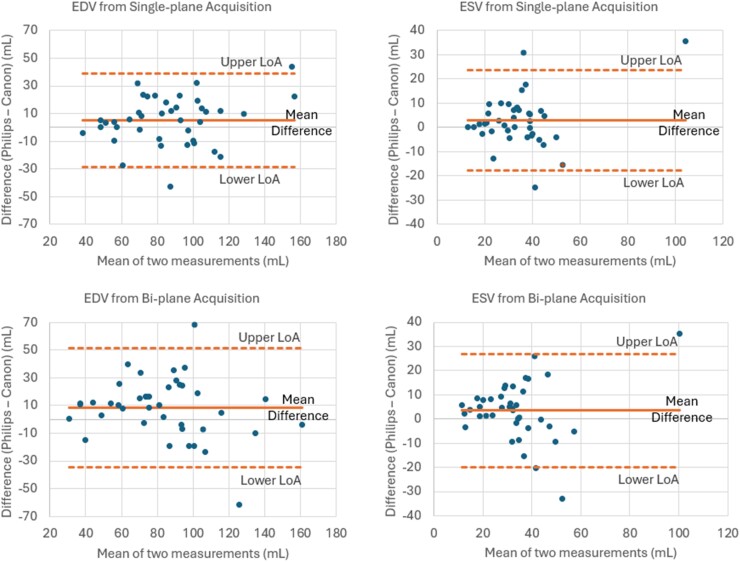
The Bland–Altman plots compare the 2D-based calculation of ventricular volumes at ED and ES. A comparison between biplane and single-plane is provided. EDV, end-diastolic volume; ESV, end-systolic volume; LoA, limit of agreement (upper is mean bias + 1.96 SD; lower is mean bias − 1.96 SD).

An example of the 2D analysis performed by Canon and Philips platforms is shown side-by-side in *Figure 1*. As given in *Table 1*, the paired *t*-tests for means reveal statistically significant differences only in ED volumes acquired by both platforms in biplane acquisitions, as indicated by the two-tailed *P*-value (*P* = 0.02). When comparing EF values, there were no statistically significant differences between the two platforms (biplane: *P* = 0.95; single-plane: *P* = 0.19). Overall, we found that the volumes reported by Canon are slightly smaller (*Table 1*) than those reported by Philips for most subjects (single-plane EDV bias = 5.19 mL or 5.25% based on the mean values, biplane EDV bias = 8.58 mL or 12.74%, single-plane ESV bias = 2.87 mL or 7.86%, biplane ESV bias = 3.46 mL or 12.62%). The correlation and Bland–Altman plots for the volumetric QoIs are provided in *Figures 2* and *3*, respectively.

**Table 1:** Comparison of cross-platform LV volumetric quantities from 2D acquisitions obtained and reported by each platform

Biplane	Philips	Canon	*P*	r	Bias	95% LOA
EDV (mL)	87.34 ± 28.98	78.76 ± 33.23	0.018	0.759	8.59 ± 21.96	−34.45 to 51.62
ESV (mL)	35.18 ± 17.34	31.72 ± 15.89	0.076	0.742	3.46 ± 12.00	−20.06 to 26.97
EF (%)	60.13 ± 9.02	60.06 ± 8.50	0.953	0.673	−0.07 ± 7.09	−13.97 to 13.84

Single-plane	Philips	Canon	*P*	r	Bias	95% LOA
EDV (mL)	89.25 ±30.01	84.07 ± 26.01	0.064	0.820	5.19 ± 17.22	−28.57 to 38.94
ESV (mL)	35.72 ± 17.16	32.85 ± 14.10	0.094	0.789	2.87 ± 10.55	−17.81 to 23.55
EF (%)	60.40 ± 7.97	61.68 ± 6.32	0.190	0.660	−1.28 ± 6.09	−13.23 to 10.66

### Volumetric and 4D strain analyses of LV and RV

**Figure 4:**
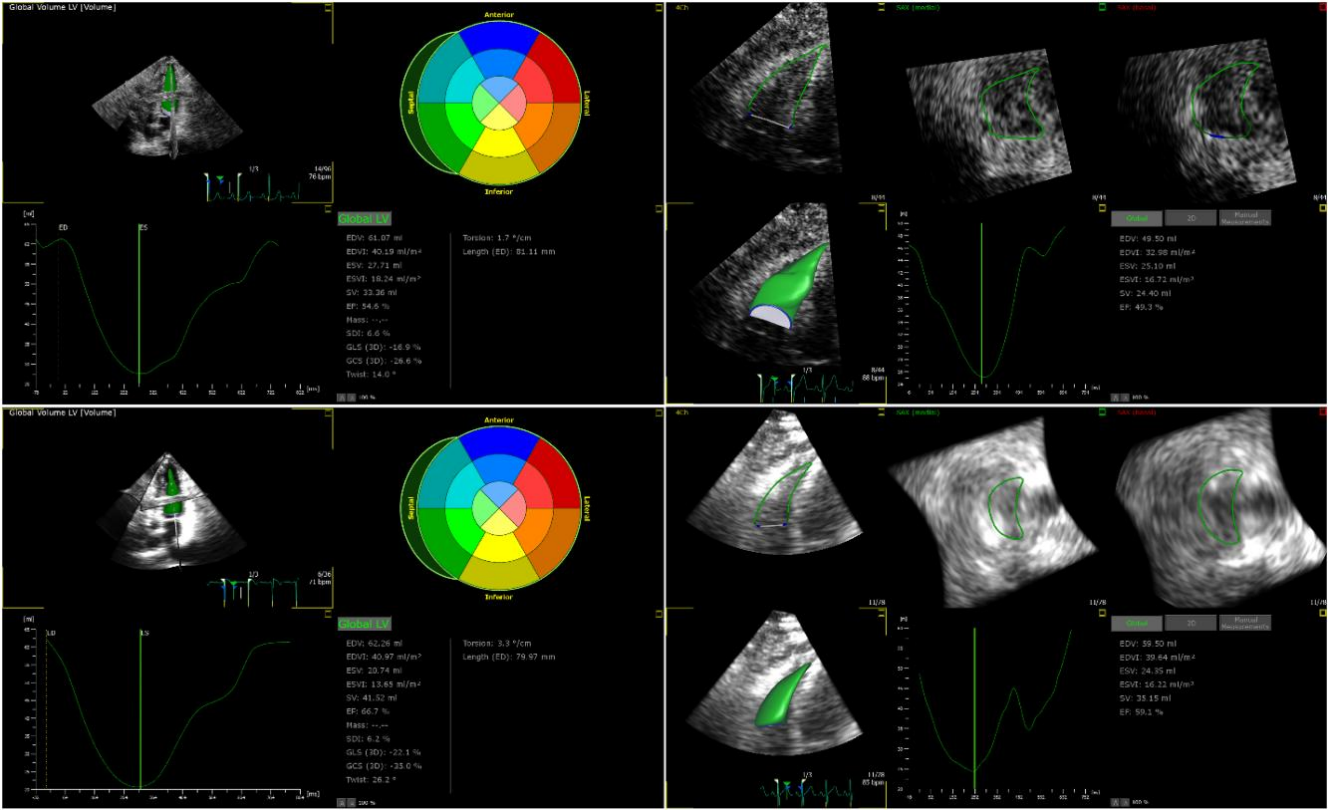
A cross-platform comparison of the 4D global strain analysis conducted by TTA for both the left and right ventricle from Philips and Canon datasets of the same subject. (Top left) 4D LV strain analysis of the Philips DICOM; (bottom left) 4D LV strain analysis of the Canon DICOM; (top right) 4D RV strain analysis of the Philips DICOM; (bottom right) 4D RV strain analysis of the Canon DICOM.

**Figure 5:**
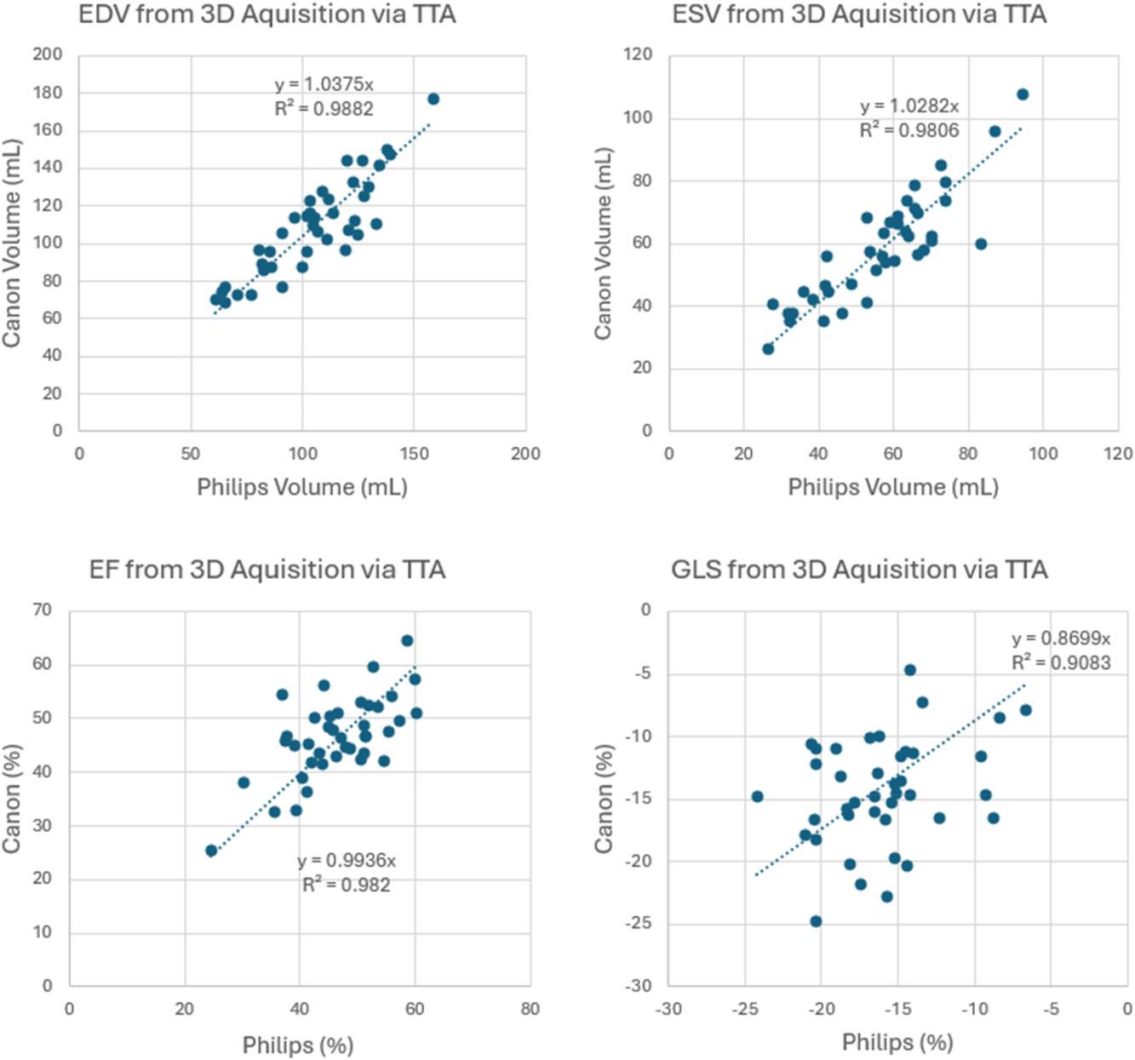
A cross-platform comparison of correlation plots depicting the 4D-acquired left ventricular results obtained from Philips and Canon systems. The correlation plots demonstrate a strong agreement between the two platforms in measuring essential parameters such as EDV, ELS, LVEF, and GLS. EDV, end-diastolic volume; ESV, end-systolic volume; LVEF, left ventricular ejection fraction; GLS, global longitudinal strain.

**Figure 6:**
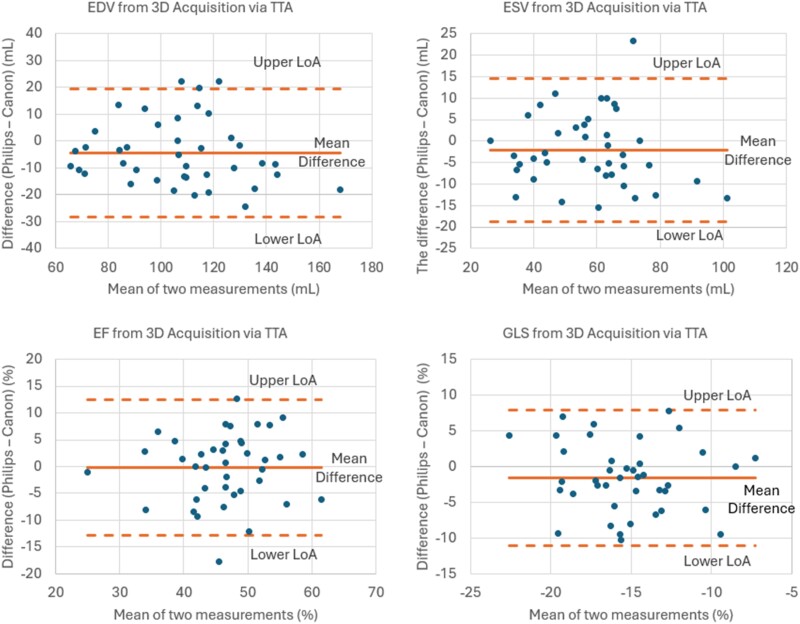
A cross-platform comparison of Bland–Altman plots depicting the 4D-acquired left ventricular results obtained from Philips and Canon systems. The correlation plots demonstrate a strong agreement between the two platforms in measuring essential parameters such as EDV, ELS, LVEF, and GLS. EDV, end-diastolic volume; ESV, end-systolic volume; LVEF, left ventricular ejection fraction; GLS, global longitudinal strain; LoA, limit of agreement (upper is mean bias + 1.96 SD; lower is mean bias − 1.96 SD).

*Figure 4* illustrates a representative example comparing the analysis conducted by TTA for both LV and RV from the same subject’s Philips and Canon datasets. Paired *t*-tests on 3D measurements in the RV and LV revealed statistically significant differences in LVEDV (*P* = 0.03), LV systolic dyssynchrony index (*P* = 0.03), LV longitudinal strain values or GLS (*P* = 0.04), LV twist (*P* = 0.00), and LV torsion (*P* = 0.01), as given in *Tables 2* and *3*. Nevertheless, as shown in *Figure 5*, LVEDV, LVESV, LVEF, and, to a lesser extent, GLS from the two platforms are strongly correlated with *R*^2^ = 0.99, 0.98, 0.99, and 0.90, respectively. The associated Bland–Altman plots for comparison and visualization of the differences are presented in *Figure 6*.

**Table 2:** Comparison of LV and RV volumetric quantities obtained from 4D acquisitions and processed with TTA Ultrasound Workspace

4D LV	Philips	Canon	*P*	r	Bias	95% LOA
EDV (mL)	104.51 ± 23.67	108.91 ± 25.43	0.028	0.877	−4.40 ± 12.18	−28.27 to 19.46
ESV (mL)	56.48 ± 16.31	58.55 ± 17.24	0.130	0.872	−2.08 ± 8.50	−18.73 to 14.58
EF (%)	46.37 ± 8.01	46.57 ± 7.53	0.840	0.660	−0.21 ± 6.42	−12.79 to 12.37

4D RV	Philips	Canon	*P*	r	Bias	95% LOA
EDV (mL)	92.17 ± 26.15	94.61 ± 23.41	0.399	0.728	−2.44 ± 18.10	−37.92 to 33.04
ESV (mL)	46.67 ± 15.32	50.49 ± 14.16	0.061	0.679	−3.62 ± 11.86	−26.87 to 19.62
EF (%)	49.51 ± 8.24	47.20 ± 7.10	0.132	0.245	2.31 ± 9.51	−16.33 to 20.96

**Table 3:** Comparison of LV strain quantities obtained from 4D acquisitions and processed with TTA Ultrasound Workspace

4D LV	Philips	Canon	*P*	r	Bias	95% LOA
GLS (%)	−16.04 ± 3.84	−14.42 ± 4.33	0.041	0.300	−1.62 ± 4.82	−11.08 to 7.85
GCS (%)	−21.2 ± 5.13	−21.38 ± 4.75	0.790	0.631	0.18 ± 4.26	−8.17 to 8.53
Twist (deg)	8.82 ± 3.85	11.65 ± 5.33	0.004	0.216	−2.83 ± 5.86	−14.32 to 8.66
Torsion (deg/mm)	1.00 ± 0.44	1.30 ± 0.60	0.005	0.271	−0.30 ± 0.64	−1.56 to 0.95
ED Length (mm)	88.64 ± 6.74	89.95 ± 7.93	0.118	0.749	−1.34 ± 5.31	−11.75 to 9.07

Other indices did not exhibit significant differences: LVESV (*P* = 0.13), LVESVI (*P* = 0.15), LVSV (*P* = 0.11), LVEF (*P* = 0.84), LV’s average circumferential strain (*P* = 0.79), LV length (*P* = 0.12), RVEDV (*P* = 0.40), RVEDVI (*P* = 0.34), RVESV (*P* = 0.06), RVESVI (*P* = 0.07), RVSV (*P* = 0.58), and RVEF (*P* = 0.13). The summary of the volumetric and strain analyses for LV and RV is given in *Tables 2* and *3*, respectively.

### Impacts on assessment of valve structure

**Figure 7:**
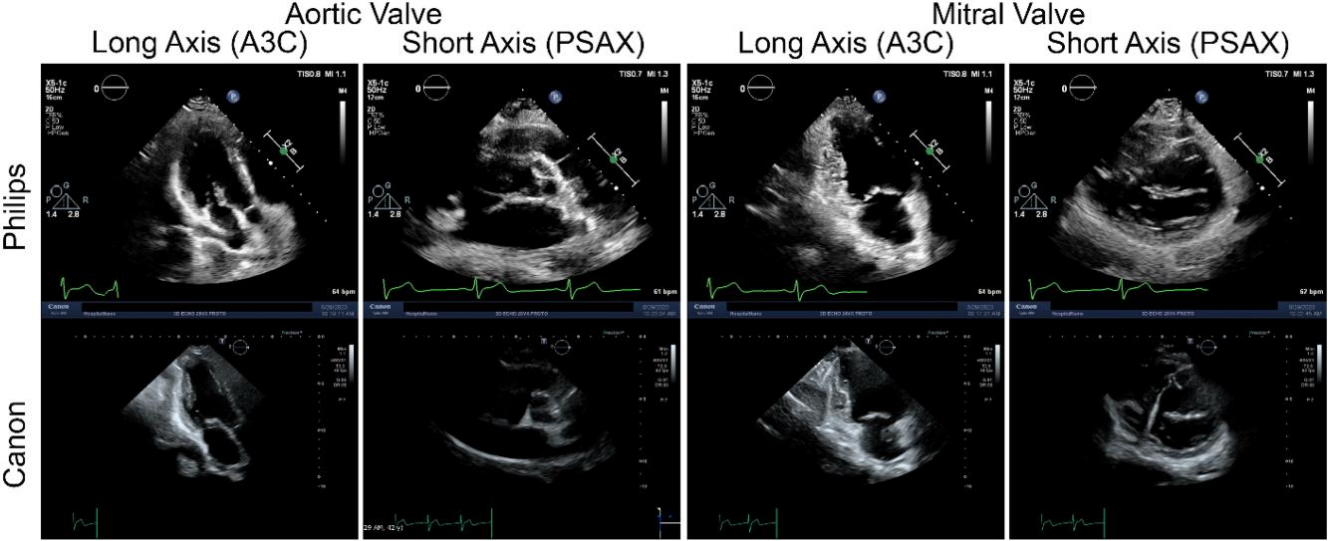
A side-by-side comparison of A3C and PSAX images acquired by each platform is presented for two representative DICOM data showing different representations of aortic and mitral valves. Comparing the DICOMS on the left-sided panels (aortic valve), MRA indicated mild calcification, and GSP suggested mild sclerosis, as per the Philips data. However, no pathology was identified based on the Canon data. Similarly, in the comparison of DICOMS on the right-sided panels (mitral valve), MRA indicated mild calcification, and GSP suggested non-specific thickening, based on the Philips data. Nonetheless, no pathology was identified based on the Canon data. Video clips are provided in the Appendix.

Upon comparing the reports individually prepared by the two cardiologists for each subject, using echocardiography data from each system, we observed that although the visual volume assessments were similar, there were discrepancies in the assessment of the aortic, mitral, and tricuspid valves for numerous subjects depending on the platform utilized. In the Philips data set, GSP diagnosed 28 subjects with mild or moderate thickening and sclerosis of the mitral valve, eight subjects with mild thickening or sclerosis of the aortic valve, and four subjects with mild thickening of the tricuspid valve. In contrast, when assessing the Canon data set, GSP diagnosed five subjects with mild or moderate thickening of the mitral valve, six subjects with mild thickening of the aortic valve, and no subjects with thickening of the tricuspid valve. When analysing the Philips data, MRA found 15 subjects with mild or moderate thickening of the mitral valve, 17 subjects with mild or moderate thickening of the aortic valve, and no subjects with thickening of the tricuspid valve. When assessing the Canon dataset MRA diagnosed only two subjects with mild or moderate thickening of the mitral valve, seven subjects with mild or moderate thickening of the aortic valve, and no subjects with thickening of the tricuspid valve. The side-by-side comparison of A3C and PSAX images acquired by each platform is presented in a representative subject in *Figure 7*.

## Discussion

This study examines the comparison of 2D and 4D echocardiographic measurements using two clinical platforms: the Philips EPIQ CVx system (version 9.0.3) and the Canon Aplio i900 system (version 7.0). The assessments were performed on a cohort of 40 volunteer subjects, with each subject being sequentially scanned by both systems by the same sonographer.

### Comparative analysis of 2D and 4D scans

Although several previous studies specified that 4D TTE is more accurate and reproducible than 2D TTE for measuring LV volumes and EF, LV quantitative analysis continues to be conducted using 2D TTE in both clinical practice and multicentre trials.^1,5,15–17^ Here, we conducted a comparison between Philips and Canon platforms to assess the agreement between their 2D TTE and 4D TTE scans in measuring LV volumes and LVEF.

In both the Canon and Philips systems, 2D LV measurements involve significant manual processing, albeit with different procedures. In the Canon system, users create a contour of the LV chamber’s endocardial border in the A2C and A4C image sets to calculate its volume. Then, the software computes the LV volume based on the user's manual input. Conversely, in the Philips system, whether using single-plane or biplane acquisition, the process begins with the user clicking on the mitral valve insertion point on the lateral wall, followed by the septal wall insertion point. The software then generates a basic model of an A2C or A4C ventricle, aligning its apex with that of the image. Subsequently, users manually adjust the contour to match the true LV endocardial border. After completing these adjustments, the software computes the LV volume based on the user’s manual inputs.

**Figure 8:**
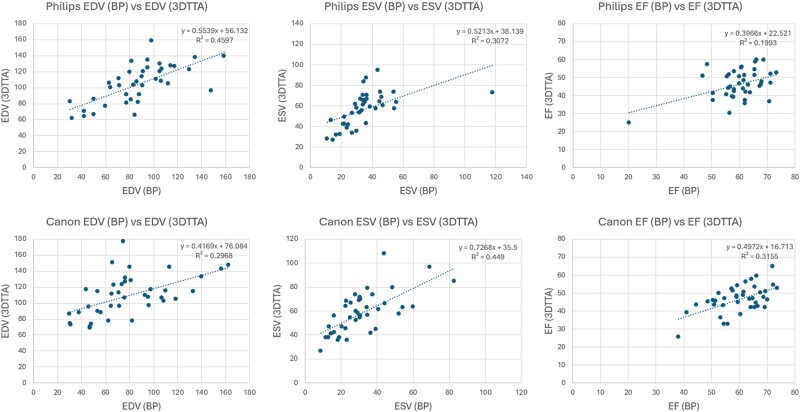
An intra-platform comparison of correlation plots between the 2D biplane and 4D acquisition methods: the correlation plots comparing left ventricular volume measurements based on 2D acquisitions using the biplane (BP) calculation method vs. 4D acquisitions employing the TomTec (3DTTA) segmentation method are depicted.

**Figure 9:**
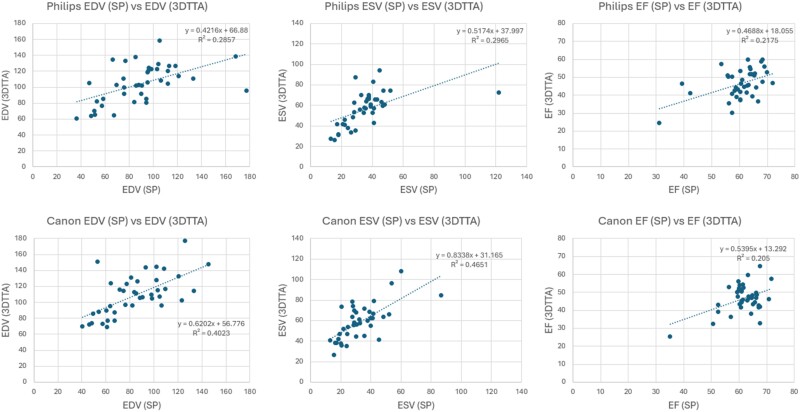
An intra-platform comparison of correlation plots between the 2D single-plane and 4D acquisition methods: the correlation plots comparing left ventricular volume measurements based on 2D acquisitions using the single-plane (SP) calculation method vs. 4D acquisitions employing the TomTec (3DTTA) segmentation method are depicted.

In both Philips and Canon studies, we observed statistically significant differences between reported volumes obtained from 2D (either biplane or single-plane) and 4D volume measurements (*Tables 4* and *5*). *Figures 8* and *9* depict intra-platform comparison of correlation plots between the 4D acquisition methods and 2D biplane and single-plane, respectively. Notably, all measured parameters exhibited correlation coefficients (*R*^2^) <0.50.

**Table 4:** Intra-platform comparison of 4D-measured LV quantities using TTA Ultrasound Workspace with biplane (BP) calculation modality for 2D-measured quantities

Philips	3D TTA	2D BP	*P*	r	Bias	95% LOA
EDV (mL)	104.51 ± 23.67	87.34 ± 28.98	1.2E-05	0.678	−17.00 ± 22.05	−60.21 to 26.21
ESV (mL)	56.48 ± 16.31	35.18 ± 17.34	2.3E-10	0.554	−21.13 ± 16.49	−53.46 to 11.20
EF (%)	46.37 ± 8.01	60.13 ± 9.02	6.6E-12	0.446	13.80 ± 9.22	−4.27 to 31.87

Canon	3D TTA	2D BP	*P*	r	Bias	95% LOA
EDV (mL)	108.91 ± 25.43	78.76 ± 33.23	7.2E-8	0.545	−30.16 ± 28.81	−86.63 ± 26.32
ESV (mL)	58.55 ± 17.24	31.72 ± 15.89	2.8E-15	0.670	−26.83 ± 13.51	−53.32 to −0.35
EF (%)	46.57 ± 7.53	60.06 ± 8.50	7.3E-14	0.660	−13.49 ± 7.55	−28.29 to 1.31

**Table 5:** Intra-platform comparison of 4D-measured LV quantities using TTA Ultrasound Workspace with single-plane (SP) calculation modality for 2D-measured quantities

Philips	3D TTA	2D SP	*P*	r	Bias	95% LOA
EDV (mL)	104.51 ± 23.67	89.26 ± 30.01	7.9E-4	0.535	−13,24 ± 25.77	−63.75 to 37.28
ESV (mL)	56.48 ± 16.31	35.72 ± 17.16	5.0E-10	0.544	−20.39 ± 16.57	−52.86 to 12.08
EF (%)	46.37 ± 8.01	60.40 ± 7.97	3.2E-13	0.466	14.73 ± 7.49	0.05 to 29.41

Canon	3D TTA	2D SP	*P*	r	Bias	95% LOA
EDV (mL)	108.91 ± 25.43	84.07 ± 26.01	1.4E-8	0.634	−24.84 ± 22.00	−67.97 to 18.28
ESV (mL)	58.55 ± 17.24	32.85 ± 14.10	2.1E-15	0.682	−25.70 ± 12.82	−50.84 to −0.57
EF (%)	46.57 ± 7.53	61.68 ± 6.32	7.9E-16	0.453	−15.11 ±7.31	−29.45 to −0.78

As presented in the Results section, most 2D comparisons show that the platforms result in statistically similar measurements of the LV volume. However, it should be noted that the 2D biplane LV measurements were taken by a complete manual processing in Canon and a semi-automated one in Phillips. Nevertheless, this difference in the platforms’ methods of biplane measurement is what clinicians must deal with in practice. Therefore, even if the vendors could help us use a new and comparable technique for 2D biplane measurements, the resultant measurements would have been irrelevant for the current clinical practices and for the purpose of our study, whether ‘current’ platforms result in similar measurements and diagnoses or not.

### Cross-platform comparison of 4D strain data

The accuracy of strain measurements heavily depends on defining cardiac time events.^18,19^ Precisely identifying ED and ES holds particular significance in segmental disease, where the timing of strain peaks is as crucial as their amplitude.^19^

Comparison of LV strain quantities obtained from 4D acquisitions is shown in *Table 3*. We found that global circumferential strain (GCS) and ED length are statistically similar between the two platforms (*P* = 0.79 and *P* = 0.12, respectively). However, the difference in global longitudinal strain (GLS), twist, and torsion between the two platforms were statistically significant (*P* = 0.04, *P* = 0.00, and *P* = 0.01, respectively).

### Impacts on clinical diagnosis

An unexpected finding was the high number of reported valvular abnormalities when using the Philips platform. There were multiple reports of valvular thickening and sclerosis that were not reproduced when assessing the same patients with the Canon platform. Further, this inconsistency in reporting was present for both independent readers. Considering that the subjects were selected from a normal population mostly without known heart disease and given that they were relatively young middle-aged individuals (mean age of 56.75 ± 11.57 years), we did not anticipate a significant number of them being diagnosed with varying degrees of valvular thickening/calcification. It is important to note that images obtained with the Philips platform were read a week apart from those obtained with the Canon machine. Thus, at the time of image interpretation, the readers did not have images from both vendors available for direct comparison. The discrepancies in reporting valve thickening and sclerosis are most likely due to inherent differences in the proprietary image processing methods used by each platform. Philips’ standard post-processing produces images with a more ‘black and white’ or ‘chiaroscuro’ effect, resulting in fewer shades of grey compared with the Canon system. Although this can enhance the definition of the endocardial–blood interface, it tends to cause valve ‘blooming’ and reduces the level of detail visible. Experienced readers can adjust for this when interpreting echocardiographic images, but less experienced readers might misinterpret the valvular anatomy. These differences between vendors must also be considered when assessing patients clinically, particularly when following up patients over time if different platforms have been used. Furthermore, for longitudinal assessments where accurate volumetric measurements are crucial, such as in cardio-oncology, it is essential to consistently use the same platform and method for analysis.

### Study limitations

Some limitations of this study include its focus on comparing only Philips and Canon systems, although few other systems are available in the clinics. Additionally, the relatively small sample size and single-centre design may limit the generalizability of the findings. Although our goal was to replicate clinical practice, we rigorously controlled several parameters to create an optimal environment for unbiased comparisons between the two platforms. Repeated scans were consistently performed by the same sonographer, and technical optimizations on both systems were overseen by a company representative during acquisitions. Due to the study design, which required each volunteer to be scanned consecutively by two systems within 90 min, we prioritized volunteer comfort. As a result, we could not assess intra-, inter-, and test/re-test variability of the measurements by sonographers. This limitation prevents us from determining whether the observed differences are due to the different imaging platforms or fall within the range of measurement reproducibility. Due to the limitations, it is important to recognize that measurement variability might be even more pronounced in real-world clinical settings. Several factors can influence the appearance of valves on echocardiography, including the distance from the transducer, frame rate, LV size and contractility, and transducer frequency. Although we controlled these variables as much as possible, the appearance of the valves varied depending on the machine used. This discrepancy is likely due to differences in the post-processing algorithms employed by each device.

## Conclusion

This study finds that the majority of LV volumetric measurements derived from 2D acquisitions (including both biplane and single-plane) obtained and reported by each platform from each study subject demonstrate statistical similarity. Similar trends are observed for most LV and RV volumetric measurements derived from 4D acquisitions. However, when comparing 4D LV strain quantities, although GCS and ED length exhibit statistical similarity between the two platforms, significant differences are noted in GLS, twist, and torsion. Intra-platform comparisons of LV quantities measured by 4D and 2D methods indicate significant differences. Last but not least, this study uncovers that clinical interpretations based on each platform may yield diverse diagnostic opinions, particularly in the characterization of heart valve structure.

## Supplementary Material

qyae097_Supplementary_Data

## Data Availability

The data underlying this article will be shared on reasonable request to the corresponding author.

## Lead author biography

Dr. Arash Kheradvar is a Professor of Biomedical Engineering and Medicine (Cardiology) at the University of California, Irvine. His research focuses on cardiovascular devices and cardiac imaging technologies. A pioneer in structural heart devices, he has developed innovative heart valve technologies and advanced imaging techniques, including vortex imaging and Volumetric Echo-PIV. Dr. Kheradvar earned his M.D. from Tehran University of Medical Sciences (2000) and a Ph.D. in Bioengineering from Caltech (2006), where he also completed postdoctoral research (2007). He is recognized as a Fellow of the American Heart Association, the American Society of Echocardiography, and the American Institute of Medical and Biological Engineering.

## References

[1] Lang RM, Badano LP, Mor-Avi V, Afilalo J, Armstrong A, Ernande L et al Recommendations for cardiac chamber quantification by echocardiography in adults: an update from the American Society of Echocardiography and the European Association of Cardiovascular Imaging. Eur Heart J Cardiovasc Imaging 2015;16:233–70. 25712077 10.1093/ehjci/jev014

[2] Galderisi M, Cosyns B, Edvardsen T, Cardim N, Delgado V, Di Salvo G et al Standardization of adult transthoracic echocardiography reporting in agreement with recent chamber quantification, diastolic function, and heart valve disease recommendations: an expert consensus document of the European Association of Cardiovascular Imaging. Eur Heart J Cardiovasc Imaging 2017;18:1301–10. 29045589 10.1093/ehjci/jex244

[3] Rudski LG, Lai WW, Afilalo J, Hua L, Handschumacher MD, Chandrasekaran K et al Guidelines for the echocardiographic assessment of the right heart in adults: a report from the American Society of Echocardiography endorsed by the European Association of Echocardiography, a registered branch of the European Society of Cardiology, and the Canadian Society of Echocardiography. J Am Soc Echocardiogr 2010;23:685–713; quiz 786–8. 20620859 10.1016/j.echo.2010.05.010

[4] Kappetein AP, Head SJ, Généreux P, Piazza N, van Mieghem NM, Blackstone EH et al Updated standardized endpoint definitions for transcatheter aortic valve implantation: the valve academic research consortium-2 consensus document (VARC-2). Eur J Cardiothorac Surg 2012;42:S45–60. 23026738 10.1093/ejcts/ezs533

[5] Muraru D, Cecchetto A, Cucchini U, Zhou X, Lang RM, Romeo G et al Intervendor consistency and accuracy of left ventricular volume measurements using three-dimensional echocardiography. J Am Soc Echocardiogr 2018;31:158–68.e1. 29229493 10.1016/j.echo.2017.10.010

[6] Badano LP, Cucchini U, Muraru D, Al Nono O, Sarais C, Iliceto S. Use of three-dimensional speckle tracking to assess left ventricular myocardial mechanics: inter-vendor consistency and reproducibility of strain measurements. Eur Heart J Cardiovasc Imaging 2013;14:285–93. 22968525 10.1093/ehjci/jes184

[7] Gayat E, Ahmad H, Weinert L, Lang RM, Mor-Avi V. Reproducibility and inter-vendor variability of left ventricular deformation measurements by three-dimensional speckle-tracking echocardiography. J Am Soc Echocardiogr 2011;24:878–85. 21645991 10.1016/j.echo.2011.04.016

[8] Guta AC, Badano LP, Ochoa-Jimenez RC, Genovese D, Previtero M, Civera S et al Three-dimensional echocardiography to assess left ventricular geometry and function. Expert Rev Cardiovasc Ther 2019;17:801–15. 31770493 10.1080/14779072.2019.1697234

[9] Muraru D, Baldea SM, Genovese D, Tomaselli M, Heilbron F, Gavazzoni M et al Association of outcome with left ventricular volumes and ejection fraction measured with two-and three-dimensional echocardiography in patients referred for routine, clinically indicated studies. Front Cardiovasc Med 2022;9:1065131. 36620642 10.3389/fcvm.2022.1065131 PMC9815115

[10] Addetia K, Badano LP, Lang RM. Routine assessment of the left ventricle. Textbook of Three-Dimensional Echocardiography 2019:53–71.

[11] Dobson R, Ghosh AK, Ky B, Marwick T, Stout M, Harkness A et al BSE and BCOS guideline for transthoracic echocardiographic assessment of adult cancer patients receiving anthracyclines and/or trastuzumab. JACC CardioOncol 2021;3:1–16. 34396303 10.1016/j.jaccao.2021.01.011 PMC8352267

[12] Lang RM, Badano LP, Mor-Avi V, Afilalo J, Armstrong A, Ernande L et al Recommendations for cardiac chamber quantification by echocardiography in adults: an update from the American Society of Echocardiography and the European Association of Cardiovascular Imaging. J Am Soc Echocardiogr 2015;28:1–39.e14. 25559473 10.1016/j.echo.2014.10.003

[13] Addetia K, Miyoshi T, Amuthan V, Citro R, Daimon M, Gutierrez Fajardo P et al Normal values of left ventricular size and function on three-dimensional echocardiography: results of the world alliance Societies of Echocardiography study. J Am Soc Echocardiogr 2022;35:449–59. 34920112 10.1016/j.echo.2021.12.004

[14] Addetia K, Miyoshi T, Amuthan V, Citro R, Daimon M, Gutierrez Fajardo P et al Normal values of three-dimensional right ventricular size and function measurements: results of the world alliance Societies of Echocardiography study. J Am Soc Echocardiogr 2023;36:858–66.e1. 37085129 10.1016/j.echo.2023.04.011

[15] Jenkins C, Bricknell K, Chan J, Hanekom L, Marwick TH. Comparison of two- and three-dimensional echocardiography with sequential magnetic resonance imaging for evaluating left ventricular volume and ejection fraction over time in patients with healed myocardial infarction. Am J Cardiol 2007;99:300–6. 17261386 10.1016/j.amjcard.2006.08.026

[16] Muraru D, Badano LP, Piccoli G, Gianfagna P, Del Mestre L, Ermacora D et al Validation of a novel automated border-detection algorithm for rapid and accurate quantitation of left ventricular volumes based on three-dimensional echocardiography. Eur J Echocardiogr 2010;11:359–68. 20042421 10.1093/ejechocard/jep217

[17] Thavendiranathan P, Liu S, Verhaert D, Calleja A, Nitinunu A, Van Houten T et al Feasibility, accuracy, and reproducibility of real-time full-volume 3D transthoracic echocardiography to measure LV volumes and systolic function: a fully automated endocardial contouring algorithm in Sinus rhythm and atrial fibrillation. JACC Cardiovasc Imaging 2012;5:239–51. 22421168 10.1016/j.jcmg.2011.12.012

[18] Mada RO, Lysyansky P, Daraban AM, Duchenne J, Voigt J-U. How to define End-diastole and end-systole?: impact of timing on strain measurements. JACC Cardiovasc Imaging 2015;8:148–57. 25577447 10.1016/j.jcmg.2014.10.010

[19] Mirea O, Pagourelias ED, Duchenne J, Bogaert J, Thomas JD, Badano LP et al Variability and reproducibility of segmental longitudinal strain measurement: a report from the EACVI-ASE Strain Standardization Task Force. JACC Cardiovasc Imaging 2018 2018;11:15–24. 10.1016/j.jcmg.2017.01.027 28528147

